# Is it time for comprehensive geriatric assessment to move beyond primary care? The case for targeting medical sub-specialty practice

**DOI:** 10.1186/s13584-017-0158-z

**Published:** 2017-06-06

**Authors:** Laura K. Byerly, G. Michael Harper

**Affiliations:** Division of Geriatrics, University of California, San Francisco and San Francisco VA Medical Center, 4150 Clement Street, 181G, San Francisco, CA 94121 USA

**Keywords:** Comprehensive geriatric assessment, Geriatric outpatient care, Geriatric oncology, Geriatric nephrology, Geriatric cardiology

## Abstract

Comprehensive geriatric assessment (CGA) as a consultative service for older adults with complex medical and psychosocial challenges has existed for decades. However, studies have often showed inconsistent acceptance and implementation of geriatric recommendations by primary care providers (PCPs) raising doubts about the overall benefits of CGA in this setting. Press and colleagues investigated the patient- and provider-related factors that affect recommendation implementation, and like previous studies, they too found similarly low rates of implementation. In this commentary, we acknowledge the perennial challenges that exist to improving the acceptance of CGA in primary care practice, and we suggest an alternative target: medical sub-specialty practice. By highlighting three medical sub-specialty fields (oncology, nephrology, and cardiology), which have demonstrated that CGA can be incorporated into their respective clinical practices, we argue that CGA may prove to have greater impact in these settings than in primary care. We also propose initial research steps that could further delineate the trends, outcomes, and next steps for such consultations.

## Background

More than 3 decades have passed since the introduction of comprehensive geriatric assessment (CGA) into the clinical literature, and yet still today, we continue to debate CGA’s value, its role in the care of older adults, and why its acceptance by primary care providers (PCPs) has been so tepid. Intended to address complex medical and psychosocial problems and their impact on a patient’s function and quality of life, CGA has at best an inconsistent record of impact on PCPs and patient outcomes [1–4]. While wide variability in how CGA is structured and delivered might confound the impact of research results, repetitive failures to show consistently high rates of recommendation implementation suggest the current approach of targeting the patients of PCPs is not consistently yielding CGA’s intended benefit [5]. Perhaps, instead, we must consider redirecting CGA to target a new audience.

In a study by Yan Press and colleagues, the very concept of *why* some patients are more likely to have CGA recommendations implemented by PCPs than others is explored [6]. These investigators retrospectively analyzed the recommendation implementation rates of over 8 years of CGA consultations to understand which factors (patient-, geriatrician-, primary physician-related) influenced the likelihood of recommendation implementation. Results were consistent with prior studies [1, 7] demonstrating low implementation rates overall; additionally, the authors demonstrated that patients with higher Charlson comorbidity index total scores had fewer PCP implemented recommendations. Press et al.’s study found no differences in recommendation implementation related to PCP demographics, rates of referrals for CGA, or the geriatricians who made the recommendations. The authors conclude that the need therefore lies in targeting ways to increase implementation rates universally at the PCP level, by equipping PCPs with better geriatric education and facilitating collaboration with consulting CGA teams.

The question, “Why do PCPs who refer patients for CGA choose to not implement more than 50% of recommendations?” is neither new to clinical practice nor to the literature. It has been discussed by many in the past without leading to sustained changes in practice [8, 9]. Press identifies that one of the issues may be the nature of PCPs’ relationships with their patients. Because of their intimate knowledge of their patients, their judgment may often trump that of the CGA team who is providing a one-time assessment. With that consideration, perhaps the broad, all-encompassing scope of recommendations from CGA in primary care settings will always have lower-yield than anticipated. The consistent finding of low recommendation implementation rates begs the question of whether primary care is the most fertile environment to demonstrate the value of CGA. If CGA recommendations could be customized for a specific clinical specialty or clinical treatment scenario, would they be received better and ultimately have more impact? Should the geriatrics community focus on a new target of non-primary care medical sub-specialists?

## An approachable target

Similar to their use in primary care settings, clinical practice guidelines in medical sub-specialty practice do not adequately address the complex medical and psychosocial needs of older adults. The wide heterogeneity in aging calls for individually tailoring sub-specialty care plans and a CGA can assist with that process. Medical sub-specialists may not have the training to address complex geriatrics issues, creating an arena for both increased need, as well as potentially increased responsiveness to CGA recommendations. While some fields have already welcomed CGA, others are just starting to recognize its potential value [5]. We will discuss below how CGA has been utilized in medical practice outside of primary care and where research could advance the future of sub-specialty clinic based CGA.

### Oncology

Oncology offers a natural opportunity for geriatrics collaboration given the complex decision making often involved and a common geriatrics goal to maximize function in older adults. Thus, oncology is one of the more studied examples of CGA in a medical sub-specialty setting [10, 11]. Several studies have demonstrated benefit of CGA embedded within oncology clinics by uncovering and optimizing medical conditions contributing to a patient’s fitness and function; assessing appropriateness of a chemotherapy regimen and adjunct non-oncologic treatments; and prioritizing patient preference and goal elicitation [11–14]. Data from Schiphorst et al. and Schulkes et al. in the Netherlands suggest that 78-93% of older oncology patients have at least 1 geriatric impairment, most of which could affect oncologic treatment plans. Remarkably, these observational studies also showed oncologists implemented CGA recommendations to treatment plans in 92-100% of patients seen [12, 14]. However, while the international oncology community acknowledges benefit from CGA, it is not yet a standard of care for complex geriatric oncology patients to undergo evaluation with CGA [10, 15].

### Nephrology

Chronic kidney disease (CKD) is a known contributor to increased rates of functional decline, frailty, and mortality in geriatric populations [16, 17]. The difficult decision making often involved in initiating dialysis, coupled with need for proper medication prescribing in the setting of impaired medication clearance makes older CKD patients a high-risk population. To date, few studies have been published highlighting CGA in outpatient nephrology clinics, but those that have, have shown promise. Hall and colleagues recently published two innovative programs designed to incorporate CGA into a nephrology clinic: one with a fellowship-trained geriatrician administering the assessment; the other utilizing the skills of a nephrologist with additional geriatric didactic training [18]. Their work found that CGA identified functional limitations in at least 25% of the elderly CKD population and that assessment led to changes in care processes in more than a third of patients, including diagnostic tests, medication changes, and follow-up consultations. Many patients were identified with cognitive impairment, chronic disease management needs, and social scenarios that warranted intervention to maximize CKD treatment or affected dialysis plans. The limited existing data suggest that CGA within a nephrology practice could provide concrete recommendations that could be directly targeted to improve the function, reduce polypharmacy, and align care plans with the goals of CKD patients.

### Cardiology

Cardiology showcases a complementary approach to CGA within its sub-specialty by using geriatrics trained cardiologists to perform CGAs. A 2011 white paper from the *Journal of the American College of Cardiology* acknowledged that geriatricians provide skills that “augment quality and capacity of cardiac specialists to meet the needs of their older patients” [19]. The expanding field of geriatric cardiology offers a CGA approach at the intersection of cardiovascular disease and geriatrics [20]. Geriatric cardiologists, though still relatively few in number and not all formally geriatrics fellowship-trained, provide CGA within a cardiology practice, selecting optimal therapeutic options based on a patient’s goals and functional status, and facilitating communication with providers [21]. Both consultative as well as longitudinal in their care approach, geriatric cardiologists offer bridges to more integrative and holistic cardiac care [20]. Given the complex co-morbidities of elderly patients with chronic cardiovascular conditions, and the growing interest in geriatric cardiology as a field, we believe there is a strong case to be made that general cardiologists will embrace CGA [21]. In a world where cardiovascular conditions dominate many geriatric patients’ problem lists, CGA on the frontlines in a cardiology sub-specialty practice might lead to a higher incidence of recommendation implementation through the collaboration between geriatricians and cardiologists, or through the rising numbers of geriatric cardiologists themselves.

## Redirecting the CGA: What are the next steps?

These three medical sub-specialties demonstrate the feasibility of integrating CGA into non-primary care spheres. But despite the above examples and evidence, a geriatrician’s perspective is not routinely sought in most medical sub-specialty practice settings. There are several possible explanations for this observation. There may be hidden boundaries that limit CGA to primary care. Perhaps the concept of CGA is poorly understood among sub-specialists or they have limited awareness of, and access to, CGA. Medical sub-specialists may not yet find the patient outcome data for CGA sufficiently convincing to routinely request it. These are hypotheses that are ripe for investigation to determine if CGA might be acceptable and ultimately effective in sub-specialty practice. Below, we outline three potential research phases that could advance our understanding of CGA and its potential applications in sub-specialty practice settings.

One of the most basic investigations for sub-specialty practice CGAs would examine what would motivate sub-specialists to seek out and follow CGA recommendations. To this end, we would ask the following questions. What are common clinical questions that sub-specialists have for geriatricians? What types of recommendations are most useful for sub-specialists and their patients? What barriers would potentially prevent implementation? Any future studies would also want to compare the rates of implemented recommendations between PCPs and sub-specialists. The answers to these research questions might highlight a fundamental difference in how the primary care and sub-specialty worlds perceive CGA. Ultimately, if we can learn *why* sub-specialists request CGA and implement its recommendations, then CGA could be redesigned to more effectively meet the needs of sub-specialists and their patients.

To successfully expand CGA to sub-specialty practice, issues of geriatrics workforce capacity will need to be addressed. Realistically, projections suggest there will not be enough fellowship-trained geriatricians in clinical practice to meet the demands of the rising number of patients requiring geriatrics expertise. This raises the question, “Do CGA practitioners need to be fellowship-trained geriatricians or can sub-specialists be taught the geriatrics knowledge and skills required to provide the key elements of CGA?” As nephrology and cardiology have shown, there is interest in adopting the latter model. Future studies would need to investigate the level of interest among sub-specialists to learn and perform CGA and the acceptance and inclusion of them within individual disciplines.

Finally, if CGA within the broad realm of sub-specialty practice proves feasible and acceptable, the next step will be to determine if it can deliver meaningful patient outcomes and potentially cost savings. Similar to Temel et al.’s work on early intervention of palliative care consultation in oncology clinics, future studies could evaluate the effect on quality of life; they could also address the professional satisfaction of sub-specialists who work with CGA teams [22]. Research could test whether CGA in sub-specialty care improves function and reduces clinical interventions for geriatric patients who are not likely to tolerate, benefit, or even potentially desire them (e.g., dialysis in a patient with end-stage renal disease). If these outcomes can be demonstrated, the additional investment needed to implement a consultative practice within sub-specialty clinics might show significant overall cost savings, as well as quality of life benefits.

## Conclusions and looking toward the future of CGA

Decades of experience with outpatient CGA targeted at PCPs and their patients has demonstrated inconsistent benefits to older patients and mixed reviews from providers. While there may still be much to learn that could lead to greater adoption of CGA recommendations in primary care, perhaps it is time to consider other applications of CGA. Since CGA attempts to incorporate a patient’s overall prognosis, functional status, goals, and comorbidities into a set of comprehensive recommendations there are many situations in sub-specialty practice where this approach could be applied. Therefore, rather than continuing to narrowly focus efforts on trying to make CGA work for PCPs and their patients, the geriatrics community should instead seek to learn if now is the time to move in a new direction. Evidence exists that CGA can be adapted to sub-specialty practice, but experience is limited and many questions remain unanswered. While the conundrum of why PCPs often elect not to implement CGA recommendations may never be solved, geriatricians can transition their efforts to a new audience that could potentially benefit patients, colleagues, and the health care system at large. CGA may never achieve universal implementation, but by identifying and focusing on the populations that would most benefit from the application of geriatric principles and the providers who are receptive to instituting those principles, the care of older patients could see marked improvement.

## Abbreviations

CGA: Comprehensive geriatric assessment
PCP: Primary care provider
CKD: Chronic kidney disease
